# Targeting the *IDH1*^R132H^ mutation in gliomas by CRISPR/Cas precision base editing

**DOI:** 10.1093/noajnl/vdae182

**Published:** 2024-11-02

**Authors:** Remi Weber, Flavio Vasella, Artsiom Klimko, Manuela Silginer, Martine Lamfers, Marian Christoph Neidert, Luca Regli, Gerald Schwank, Michael Weller

**Affiliations:** Laboratory of Molecular Neuro-Oncology, Department of Neurology, Clinical Neuroscience Centre, University Hospital and University of Zurich, Zurich, Switzerland; Department of Neurosurgery, Clinical Neuroscience Centre, University Hospital and University of Zurich, Zurich, Switzerland; Laboratory of Molecular Neuro-Oncology, Department of Neurology, Clinical Neuroscience Centre, University Hospital and University of Zurich, Zurich, Switzerland; Laboratory of Molecular Neuro-Oncology, Department of Neurology, Clinical Neuroscience Centre, University Hospital and University of Zurich, Zurich, Switzerland; Laboratory of Molecular Neuro-Oncology, Department of Neurology, Clinical Neuroscience Centre, University Hospital and University of Zurich, Zurich, Switzerland; Department of Neurosurgery, Brain Tumor Center, Erasmus University Medical Center, Rotterdam, The Netherlands; Department of Neurosurgery, Clinical Neuroscience Centre, University Hospital and University of Zurich, Zurich, Switzerland; Department of Neurosurgery, Cantonal Hospital St.Gallen, St.Gallen, Switzerland; Department of Neurosurgery, Clinical Neuroscience Centre, University Hospital and University of Zurich, Zurich, Switzerland; Laboratory of Translational Genome Editing, Institute of Pharmacology and Toxicology, University of Zurich, Zurich, Switzerland; Laboratory of Molecular Neuro-Oncology, Department of Neurology, Clinical Neuroscience Centre, University Hospital and University of Zurich, Zurich, Switzerland

**Keywords:** 2-hydroxyglutarate, AAV, CRISPR/Cas base editing, gene therapy, *IDH*-mutant glioma

## Abstract

**Background:**

Gliomas, the most frequent malignant primary brain tumors, lack curative treatments. Understanding glioma-specific molecular alterations is crucial to develop novel therapies. Among them, the biological consequences of the isocitrate dehydrogenase 1 gene mutation (*IDH1*^R132H^) remain inconclusive despite its early occurrence and widespread expression.

**Methods:**

We thus employed CRISPR/Cas adenine base editors, which allow precise base pair alterations with minimal undesirable effects, to correct the *IDH1*^R132H^ mutation.

**Results:**

Successful correction of the *IDH1*^R132H^ mutation in primary patient-derived cell models led to reduced *IDH1*^R132H^ protein levels and decreased production of 2-hydroxyglutarate, but increased proliferation. A dual adeno-associated virus split intein system was used to successfully deliver the base editor in vitro and in vivo.

**Conclusions:**

Taken together, our study provides a strategy for a precise genetic intervention to target the *IDH1*^R132H^ mutation, enabling the development of accurate models to study its impact on glioma biology and serving as a framework for an in vivo gene therapy.

**Key Points**

- An adenine base editing approach was developed to efficiently and specifically correct the *IDH1*^R132H^ mutation in different *IDH1*-mutant glioma models.
- The correction of the *IDH1*^R132H^ mutation resulted in decreased 2-hydroxyglutarate levels as well as increased proliferation in vitro.
- By packaging the adenine base editor in a split adeno-associated virus system, the *IDH1*^R132H^ mutation was corrected in patient-derived *IDH1*-mutant organoid and in vivo in mice.

**Importance of the Study**

*Isocitrate dehydrogenase* (*IDH*)-mutant gliomas are considered incurable and are associated with significant morbidity and mortality. Biologically accurate in vitro and in vivo modeling of the impact of the *IDH1*^R132H^ mutation in gliomas has proven difficult, thus representing an important limitation to further our understanding. We developed a CRISPR/Cas-based genome editing approach to specifically revert the *IDH1*^R132H^ mutation both in vitro and in vivo. Our study offers a precise genetic intervention strategy to target the *IDH1*^R132H^ mutation, providing a tool for accurate models to study glioma biology and a framework for potential gene therapy.

Gliomas are the most frequent primary malignant brain tumors and account for approximately a third of all adult brain tumors.^1^ Despite recent therapeutic advances, resistance to conventional cancer therapies as well as infiltrative growth remain hallmarks of gliomas, resulting in a lack of curative therapies and an inevitably fatal outcome.^2^

Within the relatively heterogeneous group of gliomas, molecular alterations have gained additional importance in the 2021 WHO Classification of CNS tumors. One molecular alteration with a fundamental impact on classification, present in the *isocitrate dehydrogenase (IDH) 1* or *2* genes, is the basis for distinguishing astrocytoma and oligodendroglioma from other glioma types.^3^*IDH1* encodes an enzyme that catalyzes the reversible decarboxylation of isocitrate to α-ketoglutarate in the citric acid cycle. *IDH1* mutations in gliomas almost exclusively occur at a specific arginine residue (Arg^132^). The most common *IDH1* mutation is a point mutation at position 395 where guanine is substituted by adenine (c.395G>A), resulting in an amino acid change from arginine to histidine (*IDH1*^R132H^).^4–6^ The importance of this specific mutation is further underlined by the observation that mutations in the same codon occur in a number of additional tumor types such as cholangiocarcinoma, acute myeloid leukemia, or prostate cancer, albeit at different frequencies.^7^ The recurrent and universally heterozygous mutation at the catalytic site Arg132 of IDH1 leads to an altered enzymatic activity, resulting in the production of 2-hydroxyglutarate (2-HG) which is considered a putative oncometabolite.^8^ Accordingly, IDH mutations are now universally interpreted as gain-of-function mutations.

Mutant IDH1 proteins alter cellular metabolism and, through the inhibition of histone demethylases, induce epigenetic changes.^9,10^ The ubiquitous expression of mutant *IDH1* in *IDH1*-mutant gliomas shows this to be an early event in gliomagenesis, strongly implicating *IDH1* mutations as a causative driver of glioma.^11^ In accordance with this observation, the expression of *IDH1*^R132H^ in the murine subventricular zone led to the formation of tumor-like nodules in which *IDH1*-mutant cells recapitulate features of early gliomagenesis and invade brain parenchyma.^12^ 2-HG produced by *IDH1*-mutant cells may suppress T cell function in the tumor microenvironment in a paracrine fashion, providing evidence for a nontumor cell-autonomous mechanism contributing to immune evasion.^13^ Moreover, the *IDH*-mutant genotype may have a profound impact on tryptophan metabolism, subsequently driving immunosuppressive states of intratumoral myeloid cells.^14,15^

Preclinical studies determined that the specific inhibition of mutant IDH1 using different small molecule compounds resulted in prolonged survival of xenografted mice bearing patient-derived gliomas.^16,17^ Conversely, the expression of *IDH1*^R132H^ markedly decreased proliferation in an *IDH1* wild-type (WT) glioblastoma cell line in vitro and, upon stereotactic injection in mice, prolonged survival of tumor-bearing mice in vivo.^18^ Moreover, potent antitumor effects mediated by 2-HG, directly contradicting its often-cited role as an oncometabolite, have been reported in a leukemic mouse model.^19^

In the clinic, the mutant *IDH1* inhibitor ivosidenib conferred prolonged disease control in patients with progressive or recurrent IDH-mutant gliomas, although limited to tumors lacking contrast enhancement.^20^ Similar results were obtained with vorasidenib, a brain-penetrant IDH inhibitor targeting both mutant IDH1 and IDH2 proteins.^21^ Furthermore, ivosidenib prolonged progression-free survival in a phase 3 randomized controlled trial of patients with chemotherapy-refractory *IDH1*-mutant cholangiocarcinoma.^22^ More recently, a double-blind randomized phase 3 clinical trial showed that treatment with vorasidenib improved progression-free survival in patients with newly diagnosed CNS WHO grade 2 IDH-mutant glioma without contrast enhancement.^23^

Nevertheless, while many studies concluded *IDH1*^R132H^ to be a tumor driver, others classified it as a tumor suppressor (Table 1). The conflicting conclusions drawn in the literature combined with suboptimal model systems such as overexpressing and quasi-homozygous models demand novel approaches to elucidate the impact of *IDH1*^R132H^ on glioma biology.

**Table 1.** Overview on Current Research on *IDH1*^R132H^ and Its Role as in Tumor Biology

Citation	Methodology	Conclusion
Tumor driver		
Dang et al.^8^	Analysis of 2-HG levels in *IDH1-*mutated glioma	2-HG accumulates in vivo and contributes to the formation and malignant progression of gliomas
Bardella et al.^12^	Transgenic overexpression of *IDH1*^R132H^ in mice	Overexpression of *IDH1*^R132H^ leads to the formation of tumor-like nodules
Kopinja et al.^17^	Inhibition of IDH1^R132H^ in BT142 cells (*IDH1*^R132H/-^)	IDH1^R132H^ inhibition leads to a survival benefit in mice
Jiang et al.^24^	Correlation between P53 protein and 2-HG levels	*IDH1* ^R132H^ promotes tumor formation through downregulating p53
Bunse et al.^13^	Introduction of IDH1^R132H^-expressing sarcomas in mice	2-HG suppresses T cell activity in tumors of mice
Wei et al.^25^	Introduction of the *IDH1*^R132H/WT^ mutation in astroglial cells by base editing	Cell migration upregulated, inhibited cell proliferation
Abou-Alfa et al.^22^	Phase 3 study of a targeted inhibitor (ivosidenib) of mutated IDH1 in cholangiocarcinoma	Increase in progression-free survival after IDH1^R132H^ inhibition
Mellinghoff et al.^26^	Phase 3 study of targeted inhibitor (vorasidenib) of mutated IDH1 in glioma	Increase in progression-free survival after IDH1^R132H^ inhibition
Tumor suppressor		
Bralten et al.^18^	Transgenic overexpression of IDH1^R132H^ in glioma xenograft mice	Decreased tumor proliferation and prolonged median survival observed
Núñez et al.^27^	Analysis of a genetically engineered mouse model harboring *IDH1*^R132H^	*IDH1* ^R132H^ acts as a tumor suppressor in glioma via upregulation of DNA damage response

Innovations in clustered, regularly interspaced, short palindromic repeat (CRISPR)/Cas systems have introduced a new versatile tool termed base editing. Adenine base editors (ABE) have the ability to convert an A•T base pair to a G•C base pair by deamination of adenine into inosine, which pairs with a cytosine and can subsequently be replaced by a guanine by DNA replication and repair mechanisms.^28^ Base editors have since been applied to correct disease-causing mutations in a number of in vitro and in vivo settings.^29–33^ Notably, base editing was successfully used to edit the recurrent telomerase reverse transcriptase promoter mutation, a genetic hallmark of glioblastoma, by intratumoral injection of an adeno-associated virus (AAV) type 2 vector in a mouse glioma model.^34^

## Methods

### Cell Culture

HEK-293T was obtained from Takara Bio Europe; GL-261 was obtained from the National Cancer Institute; and GS827 was generated by M. Lamfers as previously described^35^. HEK and GL-261 cells were cultured in DMEM (#11965092, ThermoFisher) with 10% FCS (#A5256701, ThermoFisher), penicillin (100 I.U./ml), and streptomycine (100 I.U./ml) (#15140122, ThermoFisher). GS827 cells were cultured in DMEM/F12 (#21331020, ThermoFisher) supplemented with GlutaMAX (#35050087, ThermoFisher), penicillin (100 I.U./ml) and streptomycine (100 I.U./ml) (#15140122, ThermoFisher), B27 (#17504044, ThermoFisher), basic fibroblast growth factor (#PHG0261, ThermoFisher) and epidermal growth factor (#PHG0311L, ThermoFisher) (both at 20 ng/ml), and heparin (5 μg/ml) (#MFCD00081689, AlfaAesar). HEK-293T was stably transduced with a 50bp fragment of the human *IDH1*^*R132H*^ gene (gatctatcatcataggtcatcatgcttatggggatcaatacagagcaact) to create the HEK reporter cell line. GL-261 cells were stably transduced with the full-length human *IDH1*^R132H^ cDNA to create GL-261-Ic.

### Base Editing

Cells were seeded at densities between 5000 to 20 000 cells/well in a 96-well plate in their respective medium 24 hours before transfection. Transfections were performed using TransIT-LT1 Transfection Reagent (#MIR2304, Mirus Bio) using 300 ng of the ABE plasmid and 100 ng of the sgRNA plasmid per well of a 96-well plate (plasmids of base editors and the sgRNAs used are described in Table 2). Transductions were performed by the addition of 10^6^ vg/cell in the respective cell medium; in the case of the organoids, the cell number was estimated based on previous measurements to be 10^6^ cells/mm^3^. For experiments where antibiotic selection was applied, cells were treated 2 days after transfection with 2 μg/ml puromycine for 3 days. Cells were harvested after 5 days (without selection) or as soon as enough cells were available (with selection). Genomic DNA was extracted from harvested cells using QuickExtract DNA Extraction Solution 1.0 (#QE09050, Lubio Science). Amplification of the *IDH1* locus was performed using a Q5 polymerase (#M0492L, New England Biolabs) and primers specific for the cDNA (f: 5’-gaccaagtcaccaaggatgc-3’, r: 5’-tgtctttaaaacgcccatca-3’) or the exogenous locus (f: 5’-ctcagagccttcgctttctg-3’, r: 5’-ccagaaatttccaacttgtatgtg-3’). Amplicons were prepared for sequencing by ExoSAP-IT™ Express PCR Product Cleanup Reagent (#75001.4X.1.ML, ThermoFisher) and sent to MicroSynth for Sanger sequencing using sequencing primers (cDNA: 5’-tgatgagaagagggttgagga-3’, exogenous DNA: 5’-gccatcactgcagttgtaggtta-3’). Sequencing files were analyzed using BEAT.^41^ To target the *IDH1* locus, the base editor was complemented with sgRNAs adapted to the protospacer adjacent motif (PAM) requirements of the individual ABEs (ataggtcatcatgcttatgg).

**Table 2.** Plasmids, ABE Constructs, and Their Respective gRNA

Name		Reference
enAs-ABE8e	5’-catcataggtcatcatgctta-3’	^36^
CP1041-ABE8e	5’-gcataggtcatcatgcttatg-3’	^36^
NG-ABE8e	5’-gataggtcatcatgcttatgg-3’	^36^
SpG-ABE8e	5’-gataggtcatcatgcttatgg-3’	^37^
ABE8e	5’-gcataggtcatcatgcttatg-3’	^36^
SaKKH-ABE8e	5’-gtcataggtcatcatgcttat-3’	^36^
ABEmaxGFP	5’-cataggtcatcatgcttatg-3’	^38^
CjCas-ABE8e	5’-gataggtcatcatgcttatgg-3’	^39^
Lenti-Guide Puro	–	^40^

### Immunoblot Analysis

Freshly harvested cells were lysed using radioimmunoprecipitation assay (RIPA) lysis buffer (#20-188, Merck Millipore) and supplemented with protease and phosphatase inhibitor cocktail (#04693132001 and #04906837001, Sigma-Aldrich). Protein concentration in the cell lysate was quantified using the Bradford protein assay (#500-0006, BioRad); 30 μg of each sample was boiled with 4x Laemmli Sample Buffer containing 10% 2-mercaptoethanol (#1610747, BioRad), run on a Mini-PROTEAN TGX Precast Gel (#4561083, BioRad), and transferred to a nitrocellulose membrane (#10600002, Sigma-Aldrich) by wet blot using the Mini Gel Tank and Blot Module (#A25977 and #B1000, ThermoFischer Scientific). The membrane was incubated with the respective antibody: anti-IDH1 R132H (Hu), (#DIA-H09, Dianova, 1:250, 4 °C overnight) followed by HRP Goat anti-mouse IgG (#405306, BioLegends, 1:2000, 20 °C 1 hour), anti-IDH1 (#ab94571, abcam, 1:1000, 4 °C overnight) followed by mouse anti-rabbit IgG-HRP (#sc-2357, 1:1000, 20 °C 1 hour) and anti-β-Actin (C4) (#sc-47778, 1:5000, 20 °C, 1 hour). Finally, proteins were visualized using the SuperSignal West Femto Maximum Sensitivity Substrate (#34095, ThermoFischer Scientific) and detected using the CURIX 60 processing system (AGFA).

### 2-HG Quantification

2-HG levels of cell lysates were quantified with the d-2-hydroxyglutarate Assay Kit (Colorimetric) (#ab211070, abcam). Cells were washed with phosphate-buffered saline (PBS), detached, washed again with PBS, lysed by 3 freeze–thaw cycles, and deproteinized with the Deproteinizing Sample Preparation Kit (#K808, BioVision). The colorimetric readout was performed using a Tecan Plate reader Infinite 200 PRO (#15058, Tecan).

### Proliferation Assay

Cells were seeded in their respective medium in a ClearView 96-well plate (#6005182, Perkin Elmer) with a quadruplicate for each time point. Imaging was started 1 day after seeding. Nuclei were stained with Hoechst 33342 (#62249, 0.2 μM, incubation for 2 hours, ThermoFisher), and 5 fields of view per well were imaged under a 4× objective in the bright and 4′,6-diamidino-2-phenylindole (DAPI) channel of a MuviCyte Live-Cell Imaging system (#HH40000000, Perkin Elmer). Cell numbers were determined by ImageJ analysis of DAPI images.

### In Vivo Experiments

All animal experiments were done in accordance with the guidelines of the Swiss federal law on animal protection and were approved by the cantonal veterinary office. C57BL/6 mice were purchased from the Charles River Laboratories. All mice were female and between 6 and 12 weeks of age. Intracranial tumor cell implantation of 100 000 GL-261-Ic cells in 2 μl was performed as previously described.^42^ Intracranial injections with the SpG-ABE8e and the GFP AAV were performed at concentrations of 4 × 10^9^ vg in 4 μl at day 5 and day 10. Brains were isolated at day 15 and either frozen in Cryochrome (#12726087, FisherScientific) or the tumor was extracted from the brain for sequencing, following the same procedure as for the organoids.

### Organoid Culture

Patient tumor tissue samples were collected at the Department of Neurosurgery, University Hospital Zurich. Informed consent was provided by all patients following the local ethical requirements and the declaration of Helsinki as well as the guidelines of the ethics committee of the canton of Zurich (KEK-ZH-Nr. 2021-00652). All the collected samples were anonymized before processing. Glioma organoids were cultured as previously described.^43^ Tissues were collected with the consent of the patients.

### Statistics

All data are represented as mean ± standard deviation of 3 replicates unless indicated differently. Statistical analyses were performed by multiple *t*-tests with the 2-stage step-up method of Benjamini, Krieger, and Yekutieli.^44^ Significance thresholds were defined as *P < .05; **P < .01; ***P < .001. Data were analyzed and visualized using Prism (GraphPad).

## Results

### Targeting the Mutant *IDH1*^R132H^ Allele by Precision Base Editing

**Figure 1.**
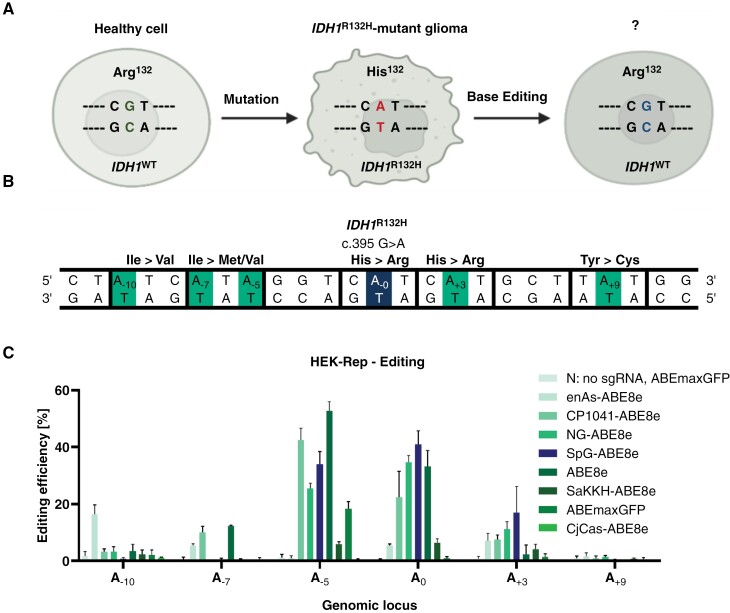
Targeting the mutant *IDH1*^*R132H*^ allele by precision base editing and selection of a suitable adenine base editor. (A) Precise and specific editing of the *IDH1*^R132H^ point mutation via adenine base editing may lead to new insights regarding its role in tumor biology. (B) Mutant *IDH1*^*R132H*^ genomic locus and editing outcome. The target base is labeled with A_0_, whereas adjacent adenines A_−10_, A_−7_, A_−5_, A_+3_, and A_+9_ may be deaminated in off-target reaction. The desired Arg > His change is achieved through A ∙ T to G ∙ C conversion of the target A_0_ which would restore *IDH1* wild-type and more importantly abrogate the neomorphic mutant enzyme activity. A ∙ T to G ∙ C conversion of A_+3_ would lead to a missense mutation and generation of an undesired Arg > His amino acid exchange at position 133. Similarly, A ∙ T to G ∙ C conversion of A_−5_ would lead also to a missense mutation (Ile > Met). (C) Editing efficiencies of the indicated base editors in different adenine positions. Experiments were performed in reporter HEK293T cells that contain the mutant IDH1^R132H^ locus.

The purpose of gene editing is the conversion of the adenine in position 395 of the *IDH1* gene into guanine, while the inevitable bystander editing is kept as low as possible (Figure 1A and B). To select an efficient ABE for the *IDH1*^R132H^ locus, a reporter HEK cell line was transfected using different constructs to identify the most suitable ABE for the *IDH1*^R132H^ locus. The highest on-target and relatively low bystander editing was achieved by SpG-ABE8e, a base editor engineered for its PAM-less characteristics^38^ (Figure 1C).

### Editing the Mutant *IDH1*^R132H^ Allele in a GL-261 Cell Model

**Figure 2.**
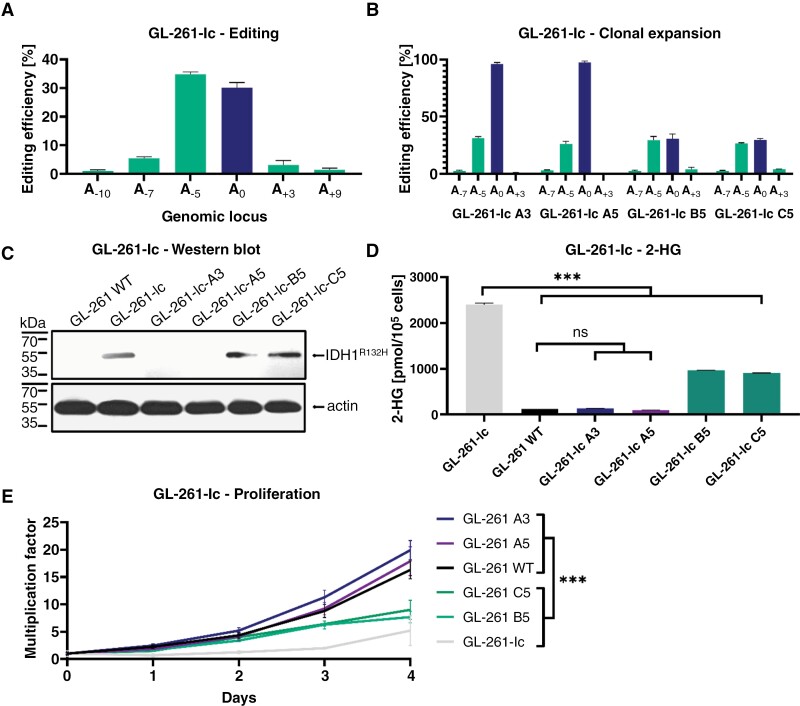
Efficient in vitro editing of the *IDH1*^*R132H*^ mutation in a murine glioma model. (A) In vitro editing efficiency in GL-261-Ic cells after plasmid-based transfection and puromycin selection. (B) Editing profiles in selected clones expanded from edited GL-261-Ic. (C) Western blot analysis of mutant IDH1 protein after gene editing. Edited and unedited cells from clonal expansions were assessed for the binding capacity of an *IDH1*^*R132H*^ mutation-specific antibody (#DIA-H09, Dianova). Beta-actin was used as a loading control. (D) Intracellular 2-HG levels of GL-261-Ic as measured by an enzymatic assay and normalized to a wild-type GL-261 lysate standard (significance levels: *P < .05; **P < .01; ***P < .001). (E) Proliferation rates of edited GL-261-Ic cells as quantified by counting of Hoechst 33342 stained nuclei over the time period of 5 days.

To study the effect of the base editor in a system with a mutant IDH1^R132H^ enzyme that could later also be applied in vivo, we generated a murine GL-261 cell line that is stably transduced with the human *IDH1*^R132H^ cDNA, referred to as GL-261-Ic (Ic: IDH1 cDNA). By plasmid-based transient transfection, we achieved editing rates of up to 30% (Figure 2A). We further performed clonal expansions to assess the phenotypic impact of editing on the cells. Two clones (A3 and A5) were edited to almost 100%, whereas 2 other clones (B5 and C5) exhibited partial editing of approximately 30% (Figure 2B).

To confirm the editing of *IDH1*^R132H^ on a protein level, we analyzed the amount of mutant protein in the cell lysate. Near-complete (A3 and A5) and partially edited clones (B5 and C5) displayed depleted or reduced levels IDH1^R132H^, indicating a genotype-dependent depletion of IDH1^R132H^ protein levels (Figure 2C). To investigate the functional impact of mutation reversal, we assessed 2-HG concentrations. To standardize the results, we established a standard of 2-HG in a GL-261 WT lysate solution. Differences in 2-HG levels were observed, indicating that the production of 2-HG was either partially or fully abrogated after editing (Figure 2D).

To assess the impact of the genetic intervention on proliferation, the cells were monitored over a 5-day period. The GL-261-Ic cells exhibited slower growth rates than cells without the *IDH1*^R132H^ mutation, comparable to the partially edited clones. However, the clones with higher editing rates (A3 and A5) demonstrated faster proliferation, similar to the GL-261-WT cells (Figure 2E). These results suggest that the transfected mutant sequence alone reduces growth rates in vitro and that editing this gene reverses this phenotype.

### Development of a Gene Therapy and Targeting of Primary Patient-Derived Tumors

**Figure 3.**
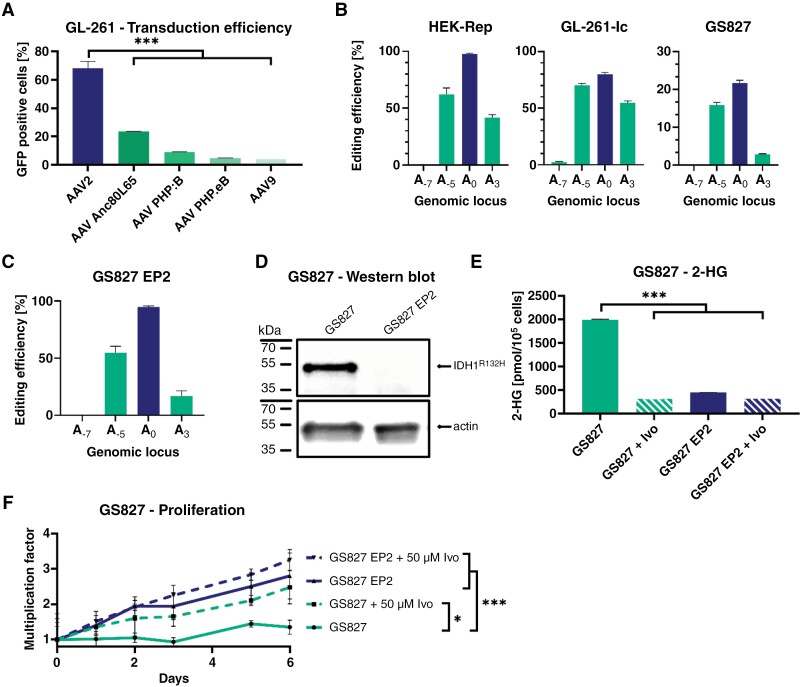
Efficient in vitro editing of the IDH1^R132H^ mutation in different glioma models and reversal of hallmark IDH1 mutant features. (A) In vitro assay to assess transduction rates of different AAV serotypes in GL261-Ic cells, as determined by flow cytometry based on transgenic GFP expression. (B) Editing rates were achieved using the intein-split base editor delivered by an AAV in all 3 cell models including primary patient-derived cells (RGI2), without antibiotic selection. (C) Editing profile of an expanded single-cell clone of the primary cell line (GS827 EP2). (D) Western blot analysis of mutant IDH1 protein after gene editing. Edited and unedited cells from clonal expansions were assessed for the binding capacity of an IDH1^R132H^ mutation-specific antibody (#DIA-H09, Dianova). Beta-actin was used as a loading control. (E) Intracellular 2-HG levels of glioma cells treated or not with ivosidenib (50 µM, 7 days), as measured by an enzymatic assay and normalized to a PBS standard.^46^ (F) Proliferation rates of edited GS827 observed by microscopy imaging over the time period of 7 days.

To allow for more efficient in vitro and in vivo delivery, we adapted the ABE by introducing a split intein site^45^ and packaging it in 2 AAV. We first determined the transduction efficiency of different AAV serotypes in GL-261 cells. The 5 AAV serotypes carrying a GFP protein were used to transduce GL-261-Ic cells which were then analyzed by flow cytometry. AAV2 reached the highest transduction efficiency of 60% and was, therefore, selected as the most suitable vector for gene therapy (Figure 3A).

We evaluated the editing efficiency by transducing 3 different cell models (HEK-Rep, GL-261-Ic, and a primary patient-derived cell line, GS827) with the virus. Higher editing rates were achieved in comparison to those achieved through plasmid-based transfection. The HEK-Rep cells were almost fully corrected in the *IDH1*^R132H^ mutation, whereas GL-261-Ic and GS827 reached editing efficiencies of 75% and 20%, respectively, without further selection (Figure 3B).

To assess the downstream effects of reversing the *IDH1*^R132H^ mutation, we expanded the edited GS827 cell line to achieve an editing rate of 100% (GS827 EP2) (Figure 3C). This led to the depletion of the IDH1^R132H^ protein (Figure 3D), and 2-HG was depleted as well, similar to the effect observed when cells were treated with an IDH1^R132H^-specific enzyme inhibitor, ivosidenib (Ivo) (Figure 3E).

To determine whether the effect observed in the GL-261-Ic model could be reproduced in patient-derived glioma cells, we performed a 7-day proliferation assay (Figure 3F). Again, *IDH1*^R132H^-mutant cells displayed slower growth compared with edited cells, comparable to the phenotype observed with pharmacological inhibition.

### Application of the *IDH1*^R132H^ Mutation Correction in Glioma Organoids and C57BL/6 Models

**Figure 4.**
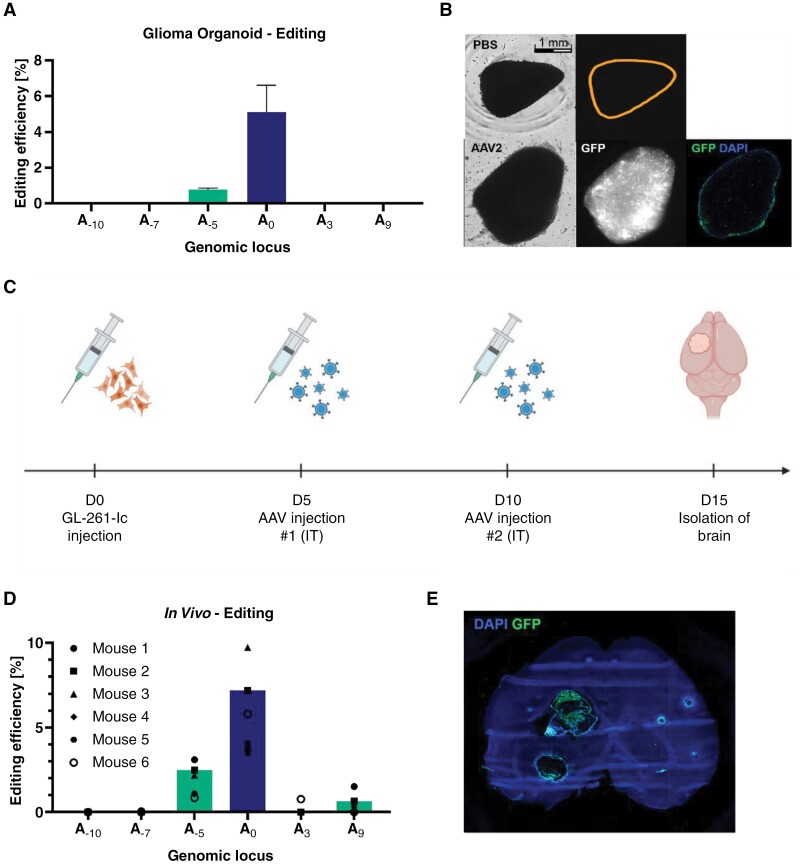
ABE-mediated editing of IDH1^R132H^ in both primary glioma organoid models and in vivo. (A) Editing efficiency in glioma organoids treated with the AAV gene therapy after two 5-day transductions, error bar represents ±SD of 2 biological replicates. (B) Representative microscopy images of glioma organoids after AAV2-GFP or PBS treatment in the bright field channel (left), the GFP channel (middle), and of a cryoslide of the organoid (right, including DAPI staining, GFP DAPI channel merge). (C) Treatment scheme for the in vivo experiment. Orthotropic GL-261-Ic tumors were treated with 4*10^9^ vg of AAV twice intratumorally (IT) with intervals of 5 days. (D) Editing rates in GL-261-Ic tumors after 2 intratumoral injections of the gene therapy. (E) Representative mouse brain cryosection 15 days after implantation of GL261-Ic and after intratumoral injection of 4*10^9^ vg of AAV2-GFP. Green channel: GFP; blue channel: DAPI.

We next aimed to correct the *IDH1* mutation in biologically more complex glioma models. After transducing primary patient-derived *IDH1*-mutant organoids^43^ twice with the AAV vector, an editing rate of 6% was achieved (Figure 4A). To investigate the reason for this low editing rate compared with adherent cell cultures, an organoid was transduced with an AAV serotype 2 packaged with GFP, whereas another organoid from the same patient was treated with PBS (Figure 4B). Although successful glioma organoid transduction could be confirmed, the majority of the GFP expression was observed at the periphery of the organoids, suggesting limited tissue penetration of the AAV.

In an in vivo study, the gene therapy was administered intratumorally twice to C57BL/6 mice carrying a GL-261-Ic tumor orthotopically (Figure 4C). Sequencing analysis showed an average editing rate of about 6% (Figure 4D). By treating a negative control group with AAV carrying GFP, we confirmed the successful transduction and expression of the AAV payload in vivo (Figure 4E).

## Discussion

*IDH1*-mutant gliomas preferentially affect young and middle-aged adults and are associated with major morbidity and mortality due to a lack of curative treatments. We here developed a precision genome editing approach to specifically correct the *IDH1*^R132H^ mutation with the aim of further assessing its role in glioma biology and its potential as a therapeutic target.

In recent years, base editing has become an increasingly attractive approach for genome editing due to its precision and ability to make genomic base substitutions without inducing double-stranded breaks. Among the ABE constructs tested, multiple displayed high A→G conversion rates at the target adenine. However, off-target edits remain a concern in base editing, and a relatively high conversion of adjacent adenines was observed when targeting the *IDH1* locus. While the SpG-ABE8e construct exhibited better editing efficiency than the other constructs tested (Figure 1C), it does display bystander editing, subsequently leading to undesired missense mutations. Although the selected base editor has the potential to yield further insights into the impact of *IDH1* in tumor biology, further refinements will be necessary to enhance its editing precision.

After selecting SpG-ABE8e as the most suitable base editor to specifically edit the *IDH1*^R132H^ mutation, its efficacy was evaluated in GL-261-Ic cells, revealing a lower editing efficiency than in HEK-Rep cells (Figure 2A). This discrepancy was attributed to the comparatively lower transfection efficiency in GL-261-Ic cells. While the cell model has important limitations relating to the overexpression of mutant IDH1 protein, it allows for the modeling of relevant aspects in *IDH*-mutant tumor biology. Notably, it can be applied in a syngeneic in vivo setting in the presence of a functional immune system. Given that immune modulation is likely to play a crucial role in glioma progression, models such as this one provide a basis for future investigations into immunobiological aspects.

Successful correction of the *IDH1*^R132H^ mutation was confirmed on both protein and functional levels in edited GL-261-Ic clones (Figure 2C and D). Of note, the near-complete reversal of the mutant genotype abrogated 2-HG production, a hallmark gain-of-function feature of the mutant enzyme. Both the pharmacologic inhibition of IDH1^R132H^ and ABE-mediated gene editing had comparable effects on 2-HG levels, showing that the genetic reversal of the point mutation abrogated the gain-of-function in the mutant enzyme.

To enable application of the ABE in primary tumor cells—which exhibit a low transfection rate when transfected with plasmids—as well as in vivo, the transduction efficiency of various AAV serotypes was assessed. AAV2, although not considered a suitable candidate for systemic gene therapies of gliomas due to its inability to cross the blood–brain barrier, demonstrated the highest transduction efficiency in vitro (Figure 3A). The significantly higher transduction rates compared with other AAV serotypes were the basis for intratumoral administration of AAV serotype 2 to bypass the blood–brain barrier for in vivo application.

The dual AAV approach led to significantly increased editing rates, likely due to a higher transduction rate relative to plasmid-based transfection. Using this approach, a patient-derived model, GS827—a human primary cell model with an endogenous *IDH1*^R132H^ mutation—was efficiently edited (Figure 3B). Moreover, repeat transduction enabled the creation of a cell model that originates from primary patient *IDH1-*mutant glioma cells converted to an *IDH1* WT status. Clonally expanded GS827 EP2 cells exhibited an increase in proliferation compared with the original *IDH1*-mutant GS827 cells, which seemingly contradicts the putative tumor-driving role of *IDH1* mutations. However, the accumulation of 2-HG caused by this mutation is known to be toxic^47^ and, therefore, may be responsible for the slower growth of *IDH1*-mutant cells. Specifically, binding and inhibition of ATP synthase by 2-HG was described to decrease mTOR signaling and subsequently suppress proliferation.^48^ A similar observation has been made, where the proliferation of astroglial cells decreased after introducing the *IDH1*^R132H^ mutation.^25^ It is conceivable that the net biological consequences of the *IDH1*^R132H^ mutation are highly context-dependent, and the mutation confers benefits outweighing the negative effects on proliferation. One possible advantage is the immunosuppressive effect of 2-HG,^13,49–51^ which could explain how the *IDH1* mutation provides a benefit to tumor growth in vivo despite decreased proliferation rates. The magnitude of any therapeutic effect achieved by pharmacologic IDH inhibition and subsequent improvement of antitumor immune response may thus be dependent on the specific tumor entity and grade, as the composition and function of immune cells within the tumor microenvironment vary between tumor grades.^52,53^ While specific clinical data serving as proof for this hypothesis are lacking, such observations are in line with the results of clinical studies, where IDH1 inhibition decreases disease progression.^21,22,54^ Whether the same benefit can be achieved in higher-grade *IDH*-mutant gliomas such as grade 4 astrocytomas is yet to be established.

Despite potentially relevant insights that could be gained from in vivo experiments examining tumorigenicity and growth dynamics of GS827 and GS827 EP2, such an approach was precluded by 2 significant constraints. Firstly, GS827 *IDH1*^R132H^ cells were not reproducibly tumorigenic in nude mice after 120 days. Secondly, the putatively important involvement of the immune compartment meant that using immunocompromised mice for xenograft experiments would not produce meaningful results.

As an alternative, the gene therapy was established in GL-261-Ic cells in C57BL/6 mice. The approach was successful in editing the *IDH1* locus, although the editing efficiency was not high enough to expect phenotypical changes. While the ABE-mediated correction of *IDH1*^R132H^ in vivo was technically feasible (Figure 4D), it is likely that editing efficiency needs to be further improved to induce meaningful phenotypical changes. While it is probable that the low editing rates observed in vivo are due to the consequence of different limitations, low efficiency of delivery is likely a crucial limiting factor. As demonstrated by the use of glioma organoids, tissue penetration of the AAV serotype 2 and thus distribution of the vector within the tumor are strongly limited, resulting in a low transduction rate (Figure 4B). In the future, advancements in both the genome editing systems themselves and the delivery methods may improve the efficiency of gene therapy in CNS tumors. The development of novel vectors displaying specifically enhanced tropism, such as evolved AAV created by directed evolution or nonviral delivery systems such as lipid-based nanoparticles, will likely contribute toward their application in CNS tumors. Moreover, promising efforts to improve drug delivery to the CNS by various means such as invasive local delivery or by transient disruption of the blood–brain barrier by focused ultrasound are being developed.^55,56^

In summary, we report the development of a highly specific genome editing approach to correct the canonical *IDH1*^R132H^ mutation. Similar approaches to correcting noncanonical *IDH1* or the canonical *IDH2*^*R172H*^ mutation could be pursued. Our study provides insights into the targeting of the *IDH1* mutation using base editing, and the resulting reversal of hallmark features both on a protein and functional level. This provides an incentive to develop novel, biologically relevant *IDH1*-mutant tumor models to further elucidate the significance of the *IDH1*^R132H^ mutation in glioma biology and assess the therapeutic potential to correct a potentially causative mutation in brain tumors. The AAV-based gene therapy demonstrates the feasibility in vivo, but underscores the need for further optimization and technological improvement.

## Data Availability

The data that support the findings of this study are available from the corresponding author upon request.
